# Social Media Use Amongst Regional Anaesthesia and Pain Practitioners and Residents: Standardization and Ethical Considerations

**DOI:** 10.4274/TJAR.2023.231211

**Published:** 2023-08-18

**Authors:** Serkan Tulgar, Ali Ahıskalıoğlu, David Terence Thomas, Alessandro De Cassai, Yavuz Gürkan

**Affiliations:** 1 Department of Anaesthesiology and Samsun University Faculty of Medicine, Samsun Training and Research Hospital, Samsun, Turkey; 2 Department of Anaesthesiology and Reanimation; Atatürk University Faculty of Medicine, Clinical Research, Development and Design Application and Research Center, Erzurum, Turkey; 3 Department of Medical Education, Maltepe University Faculty of Medicine, İstanbul, Turkey; 4 Anaesthesiology and Intensive Care, University Hospital of Padua, Padua, Italy; 5 Department of Anaesthesiology and Reanimation, Koç University Faculty of Medicine, İstanbul, Turkey

**Keywords:** Algology, ethical consideration, pain, regional anaesthesia, social media

**Dear Editor,**

Social media has become an everyday part of social interaction and communication. All parties involved in healthcare - physicians, patients, students and educators, use social media for their own benefit. Clinicians and researchers involved in regional anaesthesia and pain management, as well as other medical fields, often share their findings, achievements, personal experiences, radiological/sonographic images on social media platforms such as Twitter, Facebook, YouTube, LinkedIn, Instagram, etc. or on personal blogs.^1^

There were two exemplary studies: one which examined the use of Twitter for communication between pain physicians during Coronavirus disease-2019 (COVID-19)^2^ and one which reports changes in regional anaesthesia-related hashtags due to the COVID-19 pandemic.^3^ It is possible that, in the near future, social media will become a tool used by medical journals for the rapid dissemination of scientific information.^4^ Yet, how will the reliability of the information presented on social media be ensured, and how will ethical considerations be resolved?

Scientific information communicated through medical journals pass a rigorous review process in which scientific integrity and ethical considerations are closely scrutinized. Recently, the American Academy of Neurology (AAN) published a position statement regarding opportunities, challenges and ethical considerations for the use of social media in healthcare.^5^ Aimed at neurologists, the article includes detailed recommendations to be followed and situations to be avoided when sharing content on social media. The topic is discussed from not only the point of view of physicians but also that of patients and researchers. Ethical problems that need to be alleviated as well as suggestions about patient education, counseling and treatment are explored. The AAN suggests proper fact checking and scientific vetting of information, use of selection bias and separation of personal and professional content as some suggestions. On the other hand provision of individual medical advice over social media and discrimination on the basis of categories such as race, ethnicity, socioeconomic status, age, gender, religion, national origin, or disability are noted as some situations that need to be avoided.^5^ While suggestions made in this position statement may be a guide to regional anaesthetists and pain clinicians, it should be kept in mind that patient characteristics, respective training and professional communication significantly differ between neurology and regional anesthesiology clinics.

Amongst healthcare professionals, regional anesthesia and pain professionals are probably amongst those who most frequently use social media.^1^ Therefore the authors propose that a more comprehensive position statement for regional anesthesia and pain professionals is required.

Since there are no routine “hashtag” patterns that are standardized or recommended by medical societies, many social media messages can be left out of studies that utilize hashtags on platforms such as Twitter. Just as keywords, terms of bias, etc. are pre-determined in meta-analyses, similarly, when conducting social media research, the type of study, sample selection, and evaluation process should be standardized and pre-determined so that more rational, reliable, consistent, and repeatable studies can be conducted. Furthermore; beneficence, non-maleficence, justice, and autonomy are principles of medical ethics that healthcare professionals should pay attention to in their social media posts, as well as broadcasting ethics which is another problem that is often neglected in social media.

We believe that a recommendation; led by national anaesthesiology societies that includes experts in humanity, law, and ethics, social media consultants, and professional influencers who frequently share regional anesthesia and pain medicine-related social media posts will be useful and informative.
